# Interleukin-28B gene non-TT allele strongly predicts treatment failure for genotype 1 infected chronic hepatitis C patients with advanced fibrosis: a case control study

**DOI:** 10.1186/s12879-015-0888-x

**Published:** 2015-03-26

**Authors:** Ching-Chih Hu, Chih-Lang Lin, Liang-Che Chang, Cheng-Hung Chien, Li-Wei Chen, Ching-Jung Liu, Rong-Nan Chien

**Affiliations:** Liver Research Unit, Chang Gung Memorial Hospital, 222 Mai-Chin Road, Keelung, 20401 Taiwan; Department of Pathology, Chang Gung Memorial Hospital, 222 Mai-Chin Road, Keelung, 20401 Taiwan

**Keywords:** Advanced fibrosis, Chronic hepatitis C, Interleukin-28B, rs8099917, Sustained virologic response

## Abstract

**Background:**

The role of single nucleotide polymorphisms (SNPs) of interleukin (IL)-28B in predicting therapeutic response of pegylated interferon (peg-IFN) plus ribavirin (PR) for genotype 1 infected chronic hepatitis C patients with advanced fibrosis (AF) is limited. The aim of this study is to assess its role in predicting sustained virologic responses (SVR) to treatment.

**Methods:**

Forty-two patients with biopsy proven hepatitis C virus (HCV) related AF (group A; Ishak fibrosis score, ≥4) and 126 sex- and HCV genotype-matched patients without AF (group B; Ishak fibrosis score, ≤3) were recruited into study. All patients received PR therapy for 24 weeks. Baseline and on-treatment clinical, virological and host factors were evaluated for treatment efficacy.

**Results:**

The SVR rate was significantly lower in group A than group B patients with genotype 1 infection (24% vs. 53.3%; p = 0.011). However, it was similar in those with genotype non-1 infection (76.5% vs. 76.5%; p = 1.0). IL-28B rs8099917 genotype TT is the strongest predictor for SVR in genotype 1 infection. Patients who had TT genotype and achieved RVR in group A had similar SVR rates with those in group B (44.4% vs. 53.3%; p = 0.614). One third of patients in group A developed hematological adverse effects and had required modified doses during antiviral therapy.

**Conclusions:**

In HCV genotype 1 infected AF receiving 24 weeks of PR treatment, patients with IL28B rs8099917 genotype TT, achieving RVR had similar SVR rate with those without AF. In contrast, patients with IL-28B rs8099917 non-TT genotype without achieving RVR are suggested to stop therapy.

## Background

Chronic hepatitis C virus (HCV) infection causes important health problems worldwide and is an important cause of morbidity and mortality from sequelae such as liver cirrhosis and hepatocellular carcinoma (HCC) [1-3]. In patients with HCV-related cirrhosis, the annual incidence rate of developing hepatic decompensation and HCC are 3.9% and 1.4–8%, respectively [3-5]. The main goals of therapy in patients with HCV-related cirrhosis are to eradicate HCV, improve liver histologic activity, and reduce fibrosis. In addition, several studies have shown that chronic HCV infected patients with advanced fibrosis (AF) achieving SVR after interferon-based therapy have reduced risks of developing liver decompensation, HCC, and liver-related mortality [6-12]. Therefore, although these patients were considered to be a difficult-to-treat population with less tolerability and poor therapeutic responses to pegylated interferon plus ribavirin (PR) therapy [13,14], they could still benefit from the treatment with a lower risk of liver disease progression and liver-related complications. The data of the efficacy and safety of PR therapy in HCV-infected patients with AF [11,15-17] is limited. Therefore, we conducted this prospective study to evaluate the beneficial effect and safety of PR therapy in patients with HCV-related AF. Baseline and on-treatment clinical, virological and host factors were also evaluated for predictors of SVR.

## Methods

### Study population

From October 2003 to January 2011, we prospectively enrolled 168 consecutive treatment-naïve patients with biopsy proven chronic HCV infection into the study. AF was defined as a fibrosis score ≥4 on the Ishak modification of histologic activity index. Forty-two patients with AF were stratified as the study group (group A). Another 126 age-, sex-, and genotype-matches (1 vs. non-1) patients without AF (fibrosis scores ≤3 on the Ishak modification scores), were stratified as the control group (group B). None of the patients had received antiviral therapy before enrollment in this study. Patients with concurrent hepatitis B virus infection, toxic hepatitis, autoimmune hepatitis, primary biliary cirrhosis, or Wilson’s disease were excluded from this study. Patients with evidence of decompensated liver cirrhosis, chronic alcohol abuse, and psychiatric problems were also excluded.

All 168 patients received weekly peg-IFN injections plus daily oral RBV for 24 weeks. The prescribed pegIFN regimen consisted of pegIFN alfa-2a 180 μg or weight-based pegIFN alfa-2b 1.5 μg/kg. For genotype 1 (GT1) HCV-infected patients, the RBV dose was 1000 mg/day for those body weight (BW) <75 kg or 1200 mg for those with BW ≥ 75 kg. For genotype non-1 HCV-infected patients, the RBV dose was 800 mg/day. All patients were followed up for 24 weeks after the completion of the treatment. During the treatment period, patients had weekly outpatient visits during the first 4 weeks and visits biweekly between the 5^th^ and 24^th^ weeks. Hematological and liver biochemical tests were conducted during each visit. The HCV RNA levels were evaluated before the initiation of the treatment, at week 4, week 12, the end of the treatment (EOT) and 24 weeks after the completion of the treatment (end of follow-up; EOFU). All biochemical and virologic tests were performed in the clinical laboratories of Chang Gung Memorial Hospital. The degree of hepatic inflammation and fibrosis was graded using an Ishak modified scores and read by a single pathologist (Dr. Chang LC). Adverse events related to the PR therapy were also recorded at each visit. Patients who received more than 80% of the recommended PR dosage for more than 80% of the expected treatment period were defined as having 80/80/80 adherence [18]. Informed consent was obtained from all the patients enrolled in this study. The study was performed in accordance with the ethical guidelines of the International Conference on Harmonisation for Good Clinical Practice and has been approved by the Institutional Review Board of Chang Gung Memorial Hospital (No. 99–3012C).

### Tests for hepatitis C virus and IL-28B genotyping

Anti-HCV tests were conducted using a third-generation enzyme immunoassay kit (AxSYM® HCV Version 3.0; Abbott Laboratories, Berkshire, UK). Serum HCV RNA was quantified using a real-time polymerase chain reaction (PCR) assay (COBAS® AmpliPrep Instrument and COBAS® TaqMan® 48; Roche Molecular Systems, Inc., Branchburg, USA), with a detection limit of 15 IU/mL. HCV genotyping was determined using a linear probe assay (VERSANT™ HCV Genotype Assay (LiPA); Bayer Corporation, Tarrytown, NY, USA).

The interleukin-28B (IL-28B) gene single-nucleotide polymorphism (SNP), rs8099917 and rs12979860, were chosen according to previous reports [19-22]. The two SNPs, rs8099917 T/G and rs12979860 C/T, gene polymorphism were genotyped using PCR and specific primers as described previously [23]. The sequences were obtained from the National Center for Biotechnology Information Entrez SNP Database.

### Assessment of the efficacy of PR therapy

Rapid virologic response (RVR) was defined as undetectable serum HCV RNA by using PCR at the end of week 4 of therapy. Complete early virologic response (cEVR) was defined as undetectable serum HCV RNA at 12 weeks of therapy. The treatment efficacy was evaluated after 24 weeks of PR therapy (EOT) and after 24 weeks of follow-up (EOFU). SVR was defined as achievement of undetectable serum HCV RNA by using PCR at the EOT that was sustained after the EOFU.

### Statistical analysis

The statistic test is equivalent in form to a Chi-square test statistic. Specifically for patients with AF, a continuity corrected Chi-square test with a 0.025 two-sided significance level will have 80% power to detect a 25% difference between patients with and without AF. Assuming the test arm has a SVR rate of 44% and the control arm has a SVR rate of 70%, the sample size in each group is 36 and 109 when the allocation ratio of control arm to test arm is 3. After adjusted a 10% withdrawal rate of this study, the final sample size will be 40 and 120 of each groups.

Evaluation of the efficacy of PR therapy was assessed using intention-to-treat (ITT) analysis. For ITT analysis, patients who received more than one dose of peg-IFN were enrolled, and drug discontinuation was defined as discontinuation of the treatment without completing 80% of the expected dosage. The continuous variables were expressed as mean ± standard deviation (SD) values, and two-tailed Student’s unpaired t-test was used to evaluate the difference between the mean values. Differences between groups of categorical variables were analyzed using chi-square test or Fisher’s exact test. The significant factors were then subjected to multivariate analysis with stepwise logistic regression model to test for interactions between the different significant covariates. A p value <0.05 was considered statistically significant. The statistical analyses were performed using the SPSS ver. 12.0 software (SPSS Inc., Chicago, USA).

## Results

### Demographic and clinical characteristics

The baseline characteristics of the 168 patients are listed in Table 1. Of them, 42 (25%) with liver histology showing AF was assigned to group A. The other 126 (75%) without AF was assigned to group B. Women (57.1%) and HCV GT1 infection (59.5%) were predominant. Most of the demographic and virologic characteristics and treatment regimens were similar in both groups; however, the total bilirubin level in group A was greater than that in group B (1.2 ± 0.5 vs. 1.0 ± 0.4 mg/dL, p = 0.03). The pretreatment albumin level (3.8 ± 0.5 vs. 4.2 ± 0.4 g/dL; p = 0.001), hemoglobin level (13.5 ± 1.8 vs. 14.2 ± 1.6 g/dL; p = 0.03), and platelet counts (129.7 ± 47.3 × 10^3^ vs. 177.9 ± 56.7 × 10^3^ platelets/μL; p < 0.001) were lower in group A in comparison with those in group B. There were fewer patients with fatty liver in group A than in group B (28.6% vs. 47.6%; p = 0.03). The pretreatment viral loads of HCV genotype 1 infected patients in group A and group B are 2.32 ± 2.37×10^6^ and 2.54 ± 8.49 × 10^6^ IU/ml, respectively (p = 0.9). The pretreatment viral loads of HCV genotype non-1 infected patients in group A and group B are 1.07 ± 0.89 × 10^6^ and 1.85 ± 4.86 × 10^6^ IU/ml, respectively (p = 0.52). Among HCV genotype 1 infected patients, 10 patients (40%) in group A and 34 patients (45%) in group B had a serum HCV RNA viral load of less than 8 × 10^5^ IU/mL (p = 0.78). Among HCV genotype non-1 infected patients, 7 patients (41%) in group A and 23 patients (45%) in group B had a serum HCV RNA viral load of less than 8 × 10^5^ IU/mL (p = 0.78).There were 164 (97%) and 166 (99%) samples available for IL28B rs8099917 and rs12979860 genotyping, and the prevalence of favorable TT and CC genotype were 87% and 89%, respectively.

**Table 1:** **Patient demographic characteristics**

**Characteristic**	**Group A**	**Group B**	**p-Value**
	**(n = 42)**	**(n = 126)**	
**Age (years)** ^**†**^	60.5 ± 8.5	60.2 ± 8.4	1.0
**Male sex** ^**‡**^	18 (42.9)	34 (42.9)	1.0
**Genotype 1** ^**‡**^	25 (59.5)	75 (59.5)	1.0
**Fatty liver** ^**‡**^	12 (28.6)	60 (47.6)	0.03
**Body weight (kg)** ^**†**^	61.7 ± 7.0	61.3 ± 10.3	0.75
**Body mass index (kg/m** ^**2**^ **)** ^**†**^	24.9 ± 2.5	24.6 ± 3.2	0.59
**Necroinflammatory scores** ^**†**^	6.3 ± 2.0	6.0 ± 1.9	0.31
**Creatinine (mg/dL)** ^**†**^	0.9 ± 0.3	0.9 ± 0.6	0.81
**AST (U/L)** ^**†**^	99.2 ± 43.8	94.4 ± 62.8	0.65
**ALT (U/L)** ^**†**^	119.8 ± 62.4	137.1 ± 103.6	0.197
**AFP (ng/mL)** ^**†**^	45.5 ± 96.4	16.9 ± 42.6	0.08
**Albumin (g/dL)** ^**†**^	3.8 ± 0.5	4.2 ± 0.4	0.001
**Total bilirubin (mg/dL)** ^**†**^	1.2 ± 0.5	1.0 ± 0.4	0.03
**White blood cell s (×10** ^**3**^ **/μL)** ^**†**^	5.5 ± 1.9	5.8 ± 1.7	0.33
**Hemoglobin level (g/dL)** ^**†**^	13.5 ± 1.8	14.2 ± 1.6	0.03
**Platelet count (×10** ^**3**^ **/μL)** ^**†**^	129.6 ± 47.3	177.0 ± 54.7	<0.001
**HCV RNA (IU/mL)** ^**†**^	1.81 ± 2.00 ×10^6^	1.53 ± 6.90 ×10^6^	0.67
**Low viral load** ^**‡**^	14 (33.3)	45 (35.7)	0.78
**rs8099917 (TT)** ^**‡**^	33/40 (82.5)	110/124 (88.7)	0.307
**rs12979860 (CC)** ^**‡**^	34/40 (85)	114/126 (90.5)	0.332
**Ribavirin per body weight (mg/kg)** ^**†**^	15.7 ± 2.9	16.3 ± 2.7	0.19

HCV, hepatitis C virus; AST, aspartate aminotransferase; ALT, alanine aminotransferase; AFP, α-fetoprotein.

Low viral load, HCV RNA ≤800,000 IU/mL.

^†^mean ± SD; ^‡^no. (%).

Group A, patients with Ishak fibrosis score 4–6.

Group B, patients with Ishak fibrosis score 0–3.

### Virologic responses to treatment

After PR treatment, 67.5% and 90.9% of the patients achieved RVR and cEVR, respectively (Table 2). With respect to on-treatment responses, the RVR rates in groups A and B were similar (58.3% vs. 70.4%; p = 0.176). However, less cEVR was achieved in group A than in group B (81.1% vs. 94%; p = 0.017). After stratifying data on the basis of the HCV genotype (GT1 and non-1), the difference between these two groups was mainly observed in HCV GT1 infected patients (68.2% vs. 89.9%; p = 0.014).

**Table 2:** **Virologic responses of the different fibrosis groups**

**Response**	**Overall**	**Group A**	**Group B**	
**HCV genotype**	**n/N (%)**	**n/N (%)**	**n/N (%)**	**p-value**
**RVR**	102/151 (67.5)	21/36 (58.3)	81/115 (70.4)	0.176
** 1**	50/91 (54.9)	9/22 (40.9)	41/69 (59.4)	0.129
** Non-1**	52/60 (86.7)	12/14 (85.7)	40/46 (87)	0.9.5
**cEVR**	140/154 (90.9)	30/37 (81.1)	110/117 (94)	0.017
** 1**	77/91 (84.6)	15/22 (68.2)	62/69 (89.9)	0.014
** Non-1**	63/63 (100)	15/15 (100)	48/48 (100)	–
**ETR**	148/168 (88.1)	36/42 (85.7)	112/126 (88.9)	0.582
** 1**	84/100 (84)	21/25 (84)	63/75 (84)	1
** Non-1**	64/68 (94.1)	15/17 (88.2)	49/51 (96.1)	0.234
**SVR**	98/168 (58.3)	19/42 (45.2)	79/126 (69.7)	0.047
** 1**	46/100 (46)	6/25 (24)*	40/75 (53.3)**	0.011
** Non-1**	53/68 (76.5)	13/17 (76.5)*	39/51 (76.5)**	1.0
**SVR (with RVR)**	77/102 (75.5)	14/21 (66.7)	63/81 (77.8)	0.291
** 1**	35/50 (70)	4/9 (44.4)	31/41 (75.6)	0.065
** Non-1**	42/52 (80.8)	10/12 (83.3)	32/40 (80)	0.797
**Relapse**	50/148 (33.8)	17/36 (47.2)	33/112 (29.5)	0.05
** 1**	38/84 (45.2)^#^	15/21 (71.4)	23/63 (36.5)	0.005
** Non-1**	12/64 (18.8)^#^	2/15 (13.3)	10/49 (20.4)	0.539
**Discontinuation**	13/168 (7.7)	6/42 (14.3)	7/126 (5.6)	0.067
** 1**	8/100 (8)	3/25 (12)	5/75 (6.7)	0.395
** Non-1**	5/68 (7.4)	3/17 (17.6)	2/51 (3.9)	0.06

n, number of patients with response; N, total number of patients in the group.

HCV, hepatitis C virus; RVR, rapid virologic response; cEVR, complete early virologic response; ETR, end of treatment response; SVR, sustained virologic response.

Group A, patients with Ishak fibrosis score 4–6.

Group B, patients with Ishak fibrosis score 0–3.

*p = 0.001; **p = 0.008, ^#^p = 0.001.

At EOFU, 98 (58.3%) achieved SVR. The SVR rate was lower of patients with AF (45.2% vs. 69.7%; p = 0.047). Among patients with HCV GT1, the SVR rate was significantly lower in group A than group B (24% vs. 53.3%; p = 0.011). In contrast, in patients infected with HCV genotype non-1, the SVR rate was quite similar between these two groups (76.5% vs. 76.5%; p = 1.0). The SVR rate was substantially higher in patients infected with HCV genotype non-1 than those in GT1 in both groups (GT1 vs. non-1; 24% vs. 76.5% in group A, p = 0.001, and 53.3% vs. 76.5% in group B, p = 0.008).

The relapse rate was significantly higher among patients with HCV GT1 infection than those infected by HCV genotype non-1 (45.2% vs. 18.8%; p = 0.001). The relapse rate was also higher in group A than in group B (47.2% vs. 29.5%; p = 0.05) (Table 2). Among HCV genotype non-1 infected patients, the relapse rates were similar in both groups (13.3% vs. 20.4%; p = 0.539). However, HCV GT1-infected patients with AF showed higher relapse rates than those without AF (71.4% vs. 36.5%; p = 0.005).

### Predictors for SVR in HCV GT1 infected patients

In HCV GT1 infection, univariate analysis showed that baseline factors, younger age, male gender, pretreatment lower HCV RNA level, RVR, absence of AF, presence of fatty liver, higher alanine aminotransferase (ALT) levels, higher hemoglobin and platelet counts, and IL-28B SNP rs8099917 TT genotype, were predictors for SVR (Table 3). Multivariate analysis demonstrated that IL-28B rs8099917 genotype TT is the strongest predictor for SVR (odds ratio [OR] = 13.422; 95% confidence interval [CI], 1.277 – 141.53; p < 0.031), followed by achieving RVR (OR = 6.853; 95% CI, 2.142 – 21.931; p = 0.001), male gender (OR = 4.813; 95% CI, 1.512 – 15.324; p = 0.008), absence of AF (OR = 4.403; 95% CI, 1.166 – 16.625; p = 0.029), and younger age (OR = 0.897 per 1 year increase; 95% CI, 0.83 – 0.969; p = 0.006).

**Table 3:** **Factors predicting a sustained virologic response in HCV genotype 1-infected patients by univariate & multivariate analysis**

**Factors**	**Univariate**			**Multivariate**		
	**OR**	**95% CI**	**p-Value**	**OR**	**95% CI**	**p-Value**
**Age (years)**	0.925	0.877–0.976	0.005	0.897	0.830–0.969	0.006
**Male gender**	3.694	1.819–6.912	0.002	4.813	1.512–15.324	0.008
**Low viral load**	2.6	1.154–5.858	0.021			
**RVR**	7.233	2.84–18.422	<0.001	6.853	2.142–21.931	0.001
**No advanced fibrosis (F1-3)**	3.619	1.3–10.075	0.014	4.403	1.166–16.625	0.029
**Fatty live**	3.087	1.353–7.047	0.007			
**rs8099917 TT vs. non-TT**	13.2	1.642–106.13	0.015	13.422	1.277–141.553	0.031
**ALT (U/L)**	1.005	1.000–1.011	0.049			
**Hemoglobin (mg/dL)**	1.349	1.058–1.72	0.016			
**Platelet count (×10** ^**3**^ **/μL)**	1.012	1.003–1.021	0.007			

HCV, hepatitis C virus; OR, Odds Ratio; CI, confidence interval; Low viral load, HCV RNA ≤800,000 IU/mL; RVR, rapid virologic response; ALT, alanine aminotransferase.

### Influence of achieving RVR and IL28B rs8099917 TT genotype on SVR rates in HCV GT1 infected patients with AF

A higher SVR rate (44.4%) was obtained from HCV GT1-infected group A patients who achieved an RVR. All the nine patients achieving RVR had favorable IL28B rs8099917 TT allele. The sensitivity, specificity, positive predictive value (PPV) and negative predictive value (NPV) of achieving RVR for SVR were 67%, 69%, 44% and 85%, respectively (Table 4). With regard to IL28B rs8099917 genotype, the HCV GT1-infected patients in group A with TT allele obtained a SVR rate of 31.6%. The sensitivity, specificity, PPV and NPV of an IL28B rs8099917 TT genotype for SVR were 100%, 22%, 30% and 100%, respectively. None of the patients with rs8099917 non-TT genotype achieved an RVR and SVR.

**Table 4:** **IL28B rs8099917 TT allele and rapid virological response in predicting sustained virological response**

		**Sensitivity**	**Specificity**	**PPV**	**NPV**
**AF(+)**	**rs8099917**	100%	22%	30%	100%
	**RVR (+)**	67%	69%	44%	85%
**AF(−)**	**rs8099917**	97%	24%	59%	89%
	**RVR (+)**	80%	67%	80%	71%

AF, advanced fibrosis; RVR, rapid virologic response.

PPV, positive predictive value; NPV, negative predictive value.

### Adverse events and discontinuation of treatment

Six patients (14.3%) in group A and 7 patients (5.6%) in group B discontinued the therapy because of intolerance to treatment-related adverse effects. The withdrawal rates were slightly higher in the group A with marginal significance (14.3% in group A vs. 5.6% in group B; p = 0.067). Half of patients who discontinued the treatment did so within the first 2 months (50% vs.57%, in groups A and B, respectively). More patients with AF had reduced the regimen dose due to adverse effects (33% of group A vs. 15% of group B; p = 0.01). In our study, the main clinical adverse effects that developed during therapy included pruritus (38%), malaise (36%), insomnia (30%), anorexia (28%), dizziness (27%), fever (24%), dyspnea (23%), and myalgia (23%) (Table 5). The incidences of adverse events were similar between the 2 groups; however, more patients in group A had hematological adverse effects such as thrombocytopenia (26% of group A vs. 6% of group B; p < 0.001) and anemia (31% vs. 16%, in groups A and B, p = 0.033). Eleven patients (26%) in group A and 17 patients (13%) in group B needed erythropoietin therapy because of symptomatic anemia.

**Table 5:** **Rates of adverse events during treatment**

**Adverse effects**	**Patients with advanced fibrosis (n) (%) (N =42)**	**Patients without advanced fibrosis (n) (%) (N =126)**
**Dose modification**	14 (33)*	19 (15)*
**PegIFN**	2 (5)	2 (2)
**RBV**	12 (29)	19 (15)
**General disorder**		
Fatigue	15 (36)	47 (37)
Fever	9 (21)	31 (25)
Rigor	7 (17)	15 (12)
Headache	4 (10)	15 (12)
Asthenia	4 (10)	10 (8)
Dizziness	11 (26)	34 (27)
Weight loss	0 (0)	5 (4)
**Gastrointestinal effects**		
Abdominal pain	9 (21)	25 (20)
Anorexia	15 (36)	32 (26)
Nausea	3 (7)	10 (8)
Vomiting	0 (0)	5 (4)
Diarrhea	3 (7)	6 (5)
Constipation	1 (2)	3 (2)
**Psychiatric effects**		
Depression	2 (5)	15 (12)
Insomnia	15 (36)	35 (28)
Anxiety	2 (5)	3 (2)
**Respiratory effects**		
Cough	8 (19)	26 (21)
Dyspnea	13 (31)	25 (20)
Rhinorrhea	0 (0)	7 (6)
Sore throat	2 (5)	6 (5)
Epistaxis	0 (0)	3 (2)
**Neuromuscular effects**		
Myalgia	11 (26)	28 (22)
Arthralgia	2 (5)	6 (5)
Numbness	2 (5)	6 (5)
**Dermatologic effects**		
Pruritus	18 (43)	46 (37)
Alopecia	6 (14)	18 (14)
Rash	8 (21)	19 (15)
Dry skin	2 (5)	2 (2)
Injection site irritation	0 (0)	2 (2)
**Hematologic effects**		
Anemia^a^	13 (31)**	20 (16)**
Leukopenia^b^	5 (12)	8 (6)
Thrombocytopenia^c^	11 (26)***	7 (6)***
EPO	11 (26)	17 (14)
BT	3 (7)	5 (4)

EPO, erythropoietin; BT, blood transfusion.

*p = 0.01; **p < 0.05; ***P < 0.001.

^a^hemoglobin level <8.5 g/dL.

^b^white blood cell count <1500 cells/mm^3^.

^c^platelet count <50,000 cells/mm.

## Discussion

AF has been considered a strong negative predictor for SVR in PR combination treatment. There has been a lot of debate on the use of this combination therapy to treat patients with AF, because of a high frequency of adverse effects, a high rate of treatment discontinuation, and decreased SVR. Previous studies have shown that an overall SVR rate for patients with AF is 30–52%, but most of these results were obtained from subgroup analysis composed of only a small number of patients in large clinical trials [24,25].

Our analysis shows that HCV genotype is an important predictor for SVR. When the HCV genotypes are considered, the inferiority of treatment efficacy in patients with AF is observed only in those infected with HCV GT1 (group A vs. group B, 24.9% vs. 53.5%; p = 0.011) and not in those infected with HCV genotype non-1 (76.5% vs. 76.5%; p = 1.0). Indeed, low treatment response rates have been reported in other studies of PR treatment for HCV GT1, treatment-naive patients with AF; the SVR rates were reported to be around 15–40% [15,16,26-29]. Thus, HCV genotype non-1–infected patients with AF should be encouraged to receive the PR therapy because they seem to show an optimal response to the therapy.

Genetic polymorphism near the IL28B gene on chromosome 19 (rs 12979860 and rs8099917), encoding interferon-λ-3 (IFN-λ-3), was shown to be important predictors for SVR to PR treatment in chronic hepatitis C infection [19-22,30]. However, rare reports had studied its role in HCV-related cirrhotic patients receiving PR therapy. Shakado et al. [31] had demonstrated that patients with the rs8099917 TT allele had significant higher SVR rate than those with rs8099917 TG or GG allele (37.0% vs. 20.8%; p = 0.013). In our study, IL28B rs8099917 TT genotype was demonstrated to be the strongest predictor for SVR rate in HCV GT1–infected patient. In HCV GT1–infected patients with rs8099917 TT genotype, a SVR rate of 30% was obtained in those with AF, which was lower than that of those without AF (30% vs. 59%; p = 0.022). However, if the patients with HCV GT1 infection in group A had a rs8099917 TT genotype and achieved an RVR, a high SVR rate of 44.4% could be obtained, which was comparable to the overall SVR rates of the patients with HCV GT1 infection in group B (44.4% vs. 53.3%; p = 0.614).

In this study, we also found that none of the patients with AF and rs8099917 non-TT genotype achieved an RVR, and all of them failed to have a SVR. Thus, IL28B genotyping can be taken into consideration when determining whether a cirrhotic patient will receive PR therapy. The IL28B rs8099917 genotype had a 100% negative predictive value (NPV) of HCV GT1 infected patients with AF. The NPV of RVR is 85%. The results of our study suggested that don’t treat HCV GT1 infected patient with AF who was IL28B rs 8099917 non-TT genotype using PR reagents. In addition, the PR therapy can be stopped in HCV GT1 infected patient with AF who did not achieve an RVR during the treatment.

The combination of PR is associated with many adverse effects and is thought to be tolerated less by patients with AF than those without AF. In addition, safety is a major concern for these patients because portal hypertension-related splenomegaly increases the risk for cytopenia, including anemia, thrombocytopenia, and neutropenia [32-34]. However, in the present study, the rates of common side effects that developed during antiviral treatment were similar in patients with and without AF, except that more hematological adverse events happen to patients with AF. Those patients had more symptomatic anemia with increased need of subcutaneous erythropoietin supplement. However, the proportion of patients that discontinued therapy due to severe adverse events was similar between the 2 groups (group A vs. group B, 5% vs. 1%; p = 0.155). This findings suggest that PR treatment are well-tolerated by the chronic HCV-infected patients with AF in Taiwan.

Direct-acting antiviral agents (DAAs) are potential novel therapies that specifically target HCV (STAT-C) enzymes involved in viral replication or viral entry into the host cell (e.g., proteases and polymerases). Recent report of sofosbuvir (a kind of HCV NS5B polymerase nucleotide inhibitor) based therapy in treating cirrhotic patients has shown a 80% SVR rate at week 12 in treatment-naïve HCV GT1 infected patients when sofosbuvir is combined with pegIFN plus RBV therapy for 12 weeks [35]. Among HCV genotype-2/3 infected patients with cirrhosis, the SVR rate was 66% with 16-week sofosbuvir plus RBV combination therapy [36]. This new approach with DAAs in treating difficult-to-treat patients offers an important milestone in HCV therapy and might replace pegIFN as the mainstream of HCV treatment in the future.

The first limitation of present study is that the treatment duration in both groups is 24 weeks. This is too short in GT1 HCV-infected patients, especially in those with high viral load (>8.0 × 5 log IU/mL), regardless of reaching RVR or not. This treatment duration was stipulated in the reimbursement policy of the National Health Insurance in Taiwan at that time, but treatment duration is now determined by response to therapy. The second limitation is the small case numbers in present study, especially those with biopsy proven AF. However, in view of the indication for antiviral therapy is no longer necessary to perform liver biopsy before treatment, the present report is more important to emphasize the therapeutic efficacy and the underlying hepatic fibrosis evaluated by liver biopsy

## Conclusions

PR combination therapy is an effective treatment for HCV genotype non 1-infected patients with AF. With respect to HCV GT1–infected AF patients, showing IL-28B rs8099917 genotype TT and achieving RVR have a comparable SVR rate to those without AF. In HCV GT1 with AF, IL-28B rs8099917 non-TT genotype, PR is not the drug of choice. Waiting for DAA plus PR or IFN-free therapy is highly recommended. Furthermore, HCV GT1 patients with AF and failed to achieve an RVR, PR therapy should be stopped earlier. Otherwise, patients with AF should be encouraged to receive antiviral therapy aggressively, particularly in those with HCV genotype non-1, or GT1 with IL-28B TT allele and achieving an RVR.

## Abbreviations

IL: Interleukin
HCV: hepatitis C virus
SNP: Single nucleotide polymorphism
peg-IFN: Pegylated interferon
PR: Pegylated interferon plus ribavirin
AF: Advanced fibrosis
SVR: Sustained virologic response
RVR: Rapid virologic response
HCC: Hepatocellular carcinoma
HAI: Histologic activity index
RBV: Ribavirin
BW: Body weight
PCR: Polymerase chain reaction
cEVR: Complete early virologic response
EOT: End of treatment
EOFU: End of follow-up
ITT: Intention-to-treat
SD: Standard deviation
ALT: Alanine aminotransferase
OR: Odds ratio
CI: Confidence interval
PPV: Positive predictive value
NPV: Negative predictive value
DAA: Direct-acting antiviral agents
